# Bicarbonate Plays a Critical Role in the Generation of Cytotoxicity during SIN-1 Decomposition in Culture Medium

**DOI:** 10.1155/2012/326731

**Published:** 2012-07-09

**Authors:** Kyo Shirai, Tatsumi Okada, Kanako Konishi, Hiroshi Murata, Soichiro Akashi, Fumio Sugawara, Nobuo Watanabe, Takao Arai

**Affiliations:** Department of Applied Biological Science, Faculty of Science and Technology, Tokyo University of Science, 2641 Yamazaki, Noda, Chiba 278-8510, Japan

## Abstract

3-Morpholinosydnonimine (SIN-1) is used as a donor of peroxynitrite (ONOO^−^) in various studies. We demonstrated, however, that, the cell-culture medium remains cytotoxic to PC12 cells even after almost complete SIN-1 decomposition, suggesting that reaction product(s) in the medium, rather than ONOO^−^, exert cytotoxic effects. Here, we clarified that significant cytotoxicity persists after SIN-1 decomposes in bicarbonate, a component of the culture medium, but not in NaOH. Cytotoxic SIN-1-decomposed bicarbonate, which lacks both oxidizing and nitrosating activities, degrades to innocuous state over time. The extent of SIN-1 cytotoxicity, irrespective of its fresh or decomposed state, appears to depend on the total number of initial SIN-1 molecules per cell, rather than its concentration, and involves oxidative/nitrosative stress-related cell damage. These results suggest that, despite its low abundance, the bicarbonate-dependent cytotoxic substance that accumulates in the medium during SIN-1 breakdown is the cytotoxic entity of SIN-1.

## 1. Introduction

Nitric oxide (NO) is produced from L arginine in various tissues by NO synthases (NOSs), and it acts as a signaling molecule via several mechanisms, including activation of soluble guanylate cyclase (sGC) and S nitrosation of cysteine thiols in proteins [1–3]. However, under some pathological conditions such as inflammation, excessively produced NO and superoxide anion (O_2_^.^^−^) react with each other to form the potent oxidant peroxynitrite (ONOO^−^), which causes oxidative damage to proteins, lipids, and DNA [4, 5]. It has become increasingly evident that ONOO^−^  formation is involved in a number of pathological conditions, including atherosclerosis and neurodegenerative disorders [4, 5]. Thus, the biological effects of ONOO^−^  have become an important area of research.

**Figure 1:**
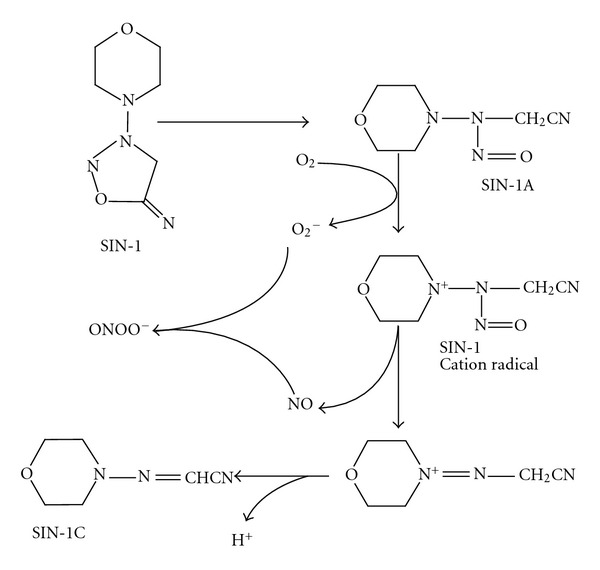
Scheme for SIN-1 decomposition and ONOO^−^ formation.

At physiological pH, ONOO^−^ undergoes protonation to form peroxynitrous acid (ONOOH), which is unstable and readily dissociates into nitrogen dioxide (NO_2_) and hydroxyl radical (^.^OH)-like species [6]. Because of the instability of ONOO^−^, 3-morpholinosydnonimine (SIN-1) is widely used as an ONOO^−^ donor for various studies (Figure 1). SIN-1 liberates O_2_^.^^−^and NO in solution with a 1 : 1 stoichiometry [7], thereby generating ONOO^−^ continuously for a certain period of time (*t*_1/2_ = approximately 30 min) [8]. In a simple aerobic aqueous solution, SIN-1 undergoes base-catalyzed hydrolysis of the sydnonimine ring, resulting in SIN-1A. SIN-1A is then oxidized by O_2_ in the solution, forming O_2_^.^^−^ and a SIN-1 cation-radical intermediate. The latter liberates NO and eventually forms the stable end product 3-morpholinoiminoacetonitrile (SIN-1C) [7]. Because the O_2_^.^^−^ and NO, thus, generated are expected to form ONOO^−^, the biological effects of SIN-1 in experiments are generally attributed to the actions of ONOO^−^.

Despite this widely held assumption, controversy exists regarding the entity responsible for the biological actions of SIN-1 under some experimental conditions [9–14]. Recently, we demonstrated that while SIN-1 almost completely decomposes in a general cell-culture medium (10% fetal calf serum in RPMI 1640, FCS/RPMI) in 2 h at 37°C under 5% CO_2_  and 95% air in dark (CO_2_ incubator), the 2 h SIN-1-decomposed medium retains the same cytotoxicity as that of freshly prepared SIN-1 in FCS/RPMI, suggesting that cytotoxic substance(s) that accumulate during SIN-1 breakdown are responsible for the cytotoxicity [8]. In fact, kinetically, the 2 h SIN-1-decomposed medium is more cytotoxic, resulting in lactate dehydrogenase (LDH) leakage approximately 2 h faster than freshly prepared SIN-1 in the medium [8]. Although the identity of the cytotoxic entity in the culture medium is unclear, it is unstable and antagonized by thiol compounds [8].

In this follow-up study, we investigated the cytotoxic entity derived from SIN-1 and its cytotoxic mechanism. We report that bicarbonate ion, which is a component of the culture medium, plays a critical role in the formation of the cytotoxic substance that remains in the medium after SIN-1 decomposition. We demonstrated that the cytotoxicity depends on the initial ratio of SIN-1 molecules to cells, rather than to the SIN-1 concentration. We have also provided evidence that the cytotoxicity of freshly prepared SIN-1 in cell-culture medium is mediated by a bicarbonate-dependent toxicant.

## 2. Materials and Methods

### 2.1. Reagents

Acetyl coenzyme A, 5,5′-dithiobis(2-nitrobenzoic acid) (DTNB), L-glutamic acid, L-glutamine, NADH, oligomycin, RPMI 1640 medium (189-02025), and 5-sulfosalicylic acid were purchased from Wako Chemicals (Osaka, Japan). 3-(4,5-Dimethyl thial-2-yl)-2,5-diphenyl-tetrazolium bromide (MTT), 1-Fluoro-2,4-dinitrobenzene (DNFB), copper (II) sulfate, L lysine, L methionine, L serine, L threonine, and glutathione (GSH) were purchased from Nacalai tesque (Kyoto, Japan). N-acetyl-L-cysteine (NAC), L-aspartic acid, ATP, L cysteine, N-ethylmaleimide (NEM), nitrate reductase, rhodamine 123 (RD), and L tryptophan were purchased from Sigma-Aldrich (St. Louis, MO, USA). Bathophenanthroline disulfonic acid and D cysteine was purchased from Tokyo Kasei Co. (Tokyo, Japan) and pyruvate kinase from Roche. 2,3-Diaminonaphthalene (DAN), diethylenetriaminepentaacetic acid (DETAPAC), (±)-(*E*)-4-ethyl-2-[(*E*)-hydroxyimino]-5-nitro-3-hexenamide (NOR-3), and SIN-1 were obtained from Dojindo (Kumamoto, Japan). Dihydrorhodamine 123 (DHR) was purchased from AAT Bioquest (Sunnyvale, CA, USA). *γ*-Glu-Glu and sodium pyruvate were purchased from MP Biomedicals (Fountain Pkwy, OH, USA). Other chemicals and salts were of analytical grade.

SIN-1 stock solutions (60 mM or 100 mM) were prepared in water, and aliquots were frozen immediately at −80°C until use. Under these conditions, SIN-1 was stable for several months, as assessed by HPLC analysis.

### 2.2. Cell Treatment with SIN-1 and Cell Viability Assays

PC12 cells (a rat pheochromocytoma cell line) were routinely maintained in DMEM containing 5% horse serum, 10% bovine calf serum, penicillin G (100 U/mL), and streptomycin (100 *μ*g/mL) in a humidified atmosphere of 5% CO_2_ at 37°C. Cells were seeded in poly-L-lysine-coated 96-well plates at a density of 3000 cells per well and treated with nerve growth factor (50 ng/mL) for 4 days in RPMI 1640 medium containing 10% FCS, penicillin G (100 U/mL), and streptomycin (100 *μ*g/mL) (FCS/RPMI). The cells were exposed to SIN-1 in various media, with or without prior decomposition, as specified for each experiment. Cell viability was evaluated by LDH leakage (%) into the medium [8]. In some experiment, cell viability was also assessed by the MTT assay [15].

For the preparation of decomposed SIN-1, SIN-1 was serially diluted in FCS/RPMI up to 1.25 mM, aliquoted into cell culture plate wells, and incubated for 2 h in a CO_2_ incubator (5% CO_2_ and 95% air with water saturation at 37°C in the dark) [8]. In some experiments, SIN-1 was serially diluted up to 1 mM in water containing 10 mM NaHCO_3_ (pH approximately 10) or 10 mM NaOH and allowed to degrade aerobically at 37°C in the dark without CO_2_ incubation. In both cases, the SIN-1-diluted medium was kept below 0.5 cm in the wells during incubation (corresponding to 200 *μ*L/well in 96-well plates). For cell treatment with decomposed SIN-1 prepared in 10 mM NaHCO_3_, the SIN-1-decomposed medium was supplemented with 1/9th volume of 10-fold-concentrated PBS containing Ca^2+^ and Mg^2+^ (1408-055; Invitrogen) and glucose, for the final concentrations of 0.9 mM Ca^2+^, 0.5 mM Mg^2+^, and 10 mM glucose. After cell treatment with the conditioned medium for 2 h, the medium was replaced with fresh FCS/RPMI, cells were incubated for an additional 22 to 24 h, and viability was assessed by the LDH assay.

### 2.3. NO Electrode

NO release from SIN-1 was measured aerobically with an NO electrode (Apollo1000; World Precision Instruments, Inc., Sarasota, FL, USA). Typically, 150 *μ*L of 1 mM SIN-1 in FCS/RPMI, with or without a previous 2 h decomposition reaction, was diluted 10-fold in prewarmed PBS at 37°C, and the NO level was monitored with the NO electrode at 37°C. The assay sample was continuously stirred with a magnetic stirrer, and 1 mM of CuSO_4_ was used to oxidize the remaining intact SIN-1. The electrode was calibrated daily according to the manufacturer's instructions.

### 2.4. DHR Oxidation and DAN Nitrosation Assays

The oxidizing ability of SIN-1 was assessed by DHR oxidation [16]. Serially diluted SIN-1 (up to 1 mM) prepared in 10 mM NaHCO_3_ was allowed to decompose as described above and then neutralized by the addition of 1/9th volume of 10-fold-concentrated PBS. Aliquots (10 *μ*L) were mixed with 190 *μ*L of DHR in 0.1 M NaP*i* (pH 7.4), yielding a final DHR-123 concentration of 10 *μ*M. The reaction mixture was incubated aerobically for 2 h in the dark, and RD fluorescence was measured using a fluorescence plate reader (Fluoro Count; Packard, Meriden, CT, USA) with excitation and emission wavelengths of 490 and 530 nm, respectively. RD concentrations were calculated from an RD standard curve.

The N-nitrosating ability of SIN-1 was assessed by the DAN assay [16]. Fresh or decomposed SIN-1 (67 *μ*L) in 10 mM NaHCO_3_, as prepared above, was mixed with 133 *μ*L of reaction mixture in 0.1 M NaP*i* (pH 7.4), giving the final concentrations of 15 *μ*M and 0.1 mM for DAN and DETAPAC, respectively. Following a 2 h incubation at 25°C under aerobic conditions in the dark, the fluorescence intensity was measured with excitation and emission wavelengths of 490 and 530 nm, respectively.

### 2.5. Nitrite and Nitrate Assays

NO_2_^−^ and NO_3_^−^ levels were determined by the Griess assay following G6P/G6PDH-coupled nitrate reductase-catalyzed NO_3_^−^ reduction [17]. Samples (35 *μ*L) were mixed with or without an equal volume of reaction constituents in 0.1 M NaP*i *(pH 7.4) for the final concentrations of 0.5 mM G6P, 0.32 U/mL G6PDH, 0.1 U/mL nitrate reductase, 6.25 *μ*M FAD, and 10 *μ*M NADPH. After incubation at 25°C for 45 min, 70 *μ*L of 1% sulfanilamide were added, followed by 70 *μ*L of 0.1% naphthyl ethylenediamine. Absorbance at 540 nm was measured with a plate reader, and NO_2_^−^ and NO_3_^−^ concentrations were calculated from standard curves prepared using NaNO_2_ and NaNO_3_, respectively.

### 2.6. GSH and Mitochondrial Enzyme Assays

Cells in 6-well plates were exposed to either fresh SIN-1 in FCS/RPMI or 2 h SIN-1-decomposed FCS/RPMI for various time periods. For the GSH assay, cells were washed with PBS, lysed in a lysis buffer (0.2% v/v Triton X-100 and 1 mM DETAPAC in PBS), and centrifuged at 10,000 ×g for 5 min. The supernatants were collected, and total GSH was measured by the GSH reductase-coupled recycling assay [18].

For mitochondrial enzyme assays, cells were harvested in 25 mM sodium phosphate buffer (pH 7.4), followed by brief sonication. Complex I activity was assessed as rotenone-inhibitable NADH oxidation with decylubiquinone [19]. Complex V activity was assessed by oligomycin-sensitive ATP hydrolysis activity and was monitored by LDH-mediated NADH oxidation by pyruvate at 340 nm in a coupling reaction for ATP regeneration catalyzed by pyruvate kinase with phosphoenol pyruvate [20]. For complex IV and citrate synthase activities, the cell sonicates, prepared as mentioned above, were solubilized with dodecyl maltoside at a detergent:protein ratio of 2 mg : 1 mg. Complex IV activity was assessed as oxidation of ferric cytochrome c at 550 nm [20]. The activity was completely sodium azide-sensitive (data not shown). Citrate synthase activity was measured by coupled DTNB reduction with coenzyme A, generated from the reaction of acetyl coenzyme A and oxaloacetate [21]. 

### 2.7. HPLC and Mass Spectrometry Analysis

Fresh or decomposed SIN-1 (1 mM) in 10 mM NaHCO_3_ was prepared as described above, and 10 *μ*L aliquots were analyzed by reverse-phase HPLC (D-7000; Hitachi, Tokyo, Japan) on a C18 column (Inertsil, ODS-3, 3 × 150 mm, 5 *μ*m particles; GL Science Inc., Tokyo, Japan) at a flow rate of 0.75 mL/min. The elution solvents were 0.1% (v/v) acetic acid in water (solvent A) and 0.1% (v/v) acetic acid in acetonitrile (solvent B), and the detection wavelength was 290 nm. After injection, the mobile phase was held in 100% solvent A for 10 min, followed by a linear gradient to 100% solvent B for 30 min. Under these conditions, intact SIN-1 eluted at 2.7 min and SIN-1C at 20 min. Identification of SIN-1C was confirmed by electrospray ionization (ESI) mass spectrometry (Bruker Esquire 3000 plus) with MH^+^ of 140.0 for SIN-1C. To quantify SIN-1 and SIN-1C in FCS/RPMI, proteins were precipitated from medium containing SIN-1 (1 mM) by the addition of an equal volume of 10% (v/v) PCA, followed by centrifugation at 10,000 ×g for 5 min. The supernatant was analyzed by HPLC as described above.

Thiol modification of GSH was assessed by HPLC following derivatization [22]. GSH (50 *μ*M) was incubated with SIN-1 (1 mM)-decomposed NaHCO_3_ or control NaHCO_3_ for 30 min. The samples were then derivatized for 30 min with 10 mM iodocetic acid and 8 mM DNFB, along with 25 *μ*M glutamylglutamine as an internal standard, followed by quenching with 20 mM L lysine. The derivatives (50 *μ*L) were separated by HPLC on an NH_2_ column (Inertsil, 3.0 × 75 mm, 5 *μ*m particles; GL Science Inc.) at a flow rate of 0.75 mL/min. The mobile phase used was 80% methanol (solvent A) and 1 : 4 (v/v) mixture of 12.2 M sodium acetate buffer (pH 4.2) and 80% methanol (solvent B). After injection, the mobile phase was maintained at 85% A and 15% B for 10 min, followed by a linear gradient to 100% B for 30 min. Dinitrophenyl derivatives were detected at 365 nm.

### 2.8. Other Assays

S alkylation of the cysteine residues in BSA was carried out by using NEM. Defatted BSA in PBS (30 mg/mL) was incubated with NEM (15 mM) at room temperature for 1 h, and excess NEM was thoroughly removed by dialysis against PBS. The thiol content of BSA before and after NEM treatment was evaluated by the DTNB method using GSH as a standard. After NEM treatment, the number of free thiols decreased from 0.37 to less than 0.02 SH per BSA molecule. Protein concentration was determined by an BCA assay kit (Thermo fisher, Rockford, USA) using BSA as a standard.

### 2.9. Statistics

Data were analyzed by the Student's *t*-test or one-way analysis of variance (ANOVA) followed by the Tukey's test. *P* < 0.05 was considered significant.

## 3. Results

### 3.1. SIN-1 Completely Decomposes in Bicarbonate in 2 h

**Figure 2:**
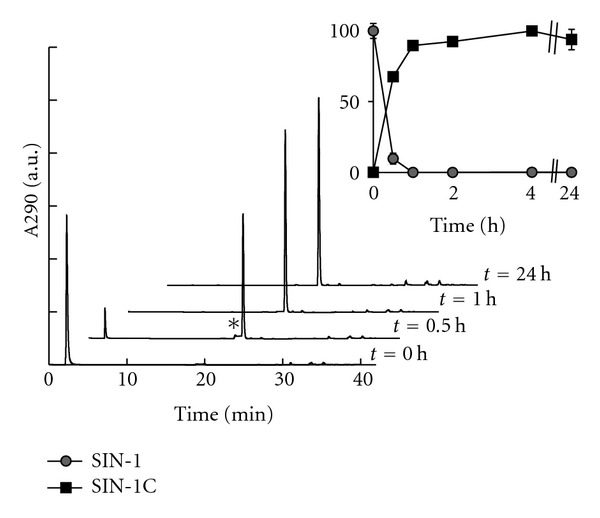
HPLC analysis of the time course of SIN-1 decomposition in bicarbonate. HPLC profile of fresh and decomposed SIN-1. SIN-1 (1 mM) dissolved in 10 mM NaHCO_3_ (pH 9.6) was allowed to decompose for up to 24 h. Aliquots were immediately separated by HPLC on a C18 column. The retention times for SIN-1 and SIN-1C were 3 min and 20 min, respectively. The asterisk indicates the possible peak for SIN-1A, which was detectable immediately before the SIN-1C peak only within the first 1 h. The inset graph shows the relative amounts of SIN-1 and SIN-1C at each time point. Areas corresponding to SIN-1 were normalized to that for *t* = 0, and the areas for SIN-1C were normalized to that obtained for 4-h-decomposed SIN-1. Values are means ± ranges of two independent experiments.

Our previous study demonstrated that SIN-1 that had been decomposed in FCS/RPMI for 2 h exhibited cytotoxicity to PC12 cells to the same extent as that by freshly prepared SIN-1 in FCS/RPMI [8]. To gain insight into the mechanism of decomposed SIN-1 cytotoxicity, we assessed the cytotoxicity of SIN-1 after decomposition in a simple solution. Because SIN-1 decomposition requires base-catalyzed hydrolysis of the sydnonimine ring as the initial step, we employed 10 mM NaHCO_3_ solution (pH 9.6) so that SIN-1 underwent facile decomposition. After aerobic incubation in 10 mM NaHCO_3_ at 37°C for various time periods, the aliquots were analyzed by HPLC to monitor the extent of decomposition (Figure 2). Without incubation, a peak corresponding to intact SIN-1 appeared at about 3 min under our analytical conditions immediately after the flow-through fraction. After initiating incubation, more than 90% of the SIN-1 disappeared within 30 min, and essentially no intact SIN-1 was detected at 1 h. Instead, a novel peak appeared at 20 min, which gradually increased over 4 h and then decreased slightly over the 24 h incubation period. The ESI mass spectrum (positive ion mode) of the elution fractions for the 20-min peak at 4 h and 24 h exhibited a cation peak at m/z 140 ([M + H]^+^), confirming it as SIN-1C (data not shown). In the earlier time period (≤1 h) only, another peak, presumably SIN-1A, was detectable at 19 min, immediately before the SIN-1C peak. Its level was highest at 30 min and had completely disappeared at 2 h.

### 3.2. Significant SIN-1 Toxicity Remains after Decomposition in NaHCO_3_, but Not in NaOH

**Figure 3:**
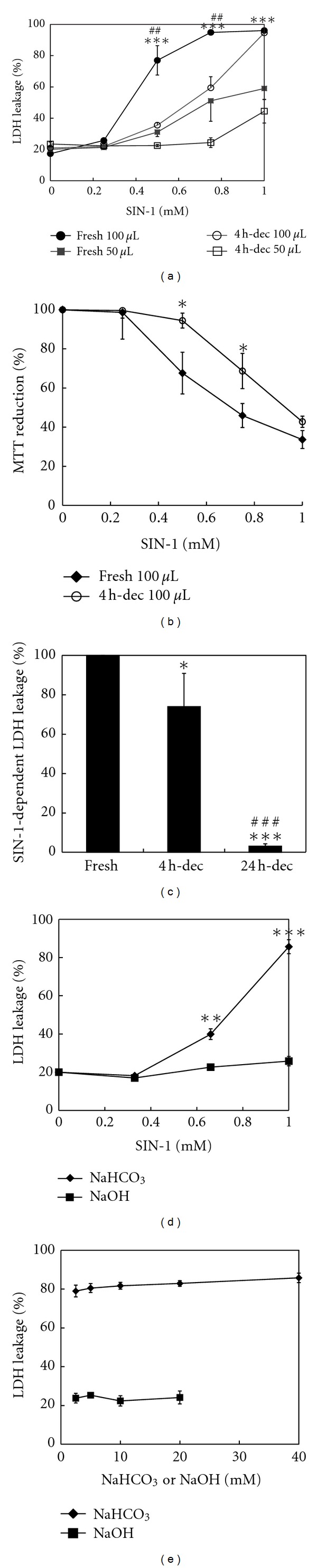
SIN-1 cytotoxicity to PC12 cells after its complete decomposition in bicarbonate. Serially diluted SIN-1 in 10 mM NaHCO_3_, either freshly prepared or allowed to decompose in advance for 4 h, was supplemented with glucose and applied to cells in 96-well plates (3000 cells/well) for 2 h. The cells were then incubated with fresh FCS/RPMI for an additional 24 h, and viability was assessed. (a) Comparison of the cytotoxicity of fresh SIN-1 and 4-h-decomposed SIN-1 in 10 mM NaHCO_3_ at 50 *μ*L/well or 100 *μ*L/well. (b) Evaluation of the cytotoxic potency of decomposed SIN-1 by MTT assay. Cells were treated with fresh SIN-1 or 4-h decomposed SIN-1 in 10 mM NaHCO_3_ as in (a), and cell viability was assessed by MTT reduction activity. (c) Decline in cytotoxicity of decomposed SIN-1. Fresh or 4-h- or 24-h-decomposed SIN-1 (1 mM)-dependent LDH leakage measured in (a) was normalized to fresh SIN-1-dependent leakage as 100%. Values are means ± inter-assay deviations, expressed as SD, from six (control and 4-h) or three (24-h) independent experiments. (d) Comparison of the cytotoxicity of 4-h-decomposed SIN-1 prepared in 10 mM NaHCO_3_ and in 10 mM NaOH. (e) Concentration dependence of NaHCO_3_ on decomposed SIN-1 cytotoxicity. SIN-1 (1 mM) was decomposed in the indicated concentrations of NaHCO_3_ or NaOH for 4 h, and cytotoxicity was measured as in (a). Unless otherwise specified, all the values are means ± intra-assay deviations, expressed as SD, from four or five wells in a representative experiment. *P < 0.05, **P < 0.01 and ***P < 0.001 versus respective control. In (a) ^##^ *P* < 0.01 versus respective 50 *μ*L/well, and in (c) ^###^ *P* < 0.001 versus 4-h-decomposed SIN-1.

We next assessed the cytotoxicity of SIN-1 in NaHCO_3_ with or without a previous 4 h decomposition reaction. The SIN-1-decomposed NaHCO_3_ solutions were neutralized with PBS, supplemented with glucose, and applied to cells. After a 2 h exposure, the medium was replaced with fresh FCS/RPMI, and viability was measured 24 h later. As shown in Figure 3(a), despite its complete decomposition within 2 h (Figure 2), the 4-h-decomposed SIN-1 exerted significant cytotoxicity that was comparable to approximately 50% of freshly prepared SIN-1; the LD_50_s for fresh and 4 h decomposed SIN-1 at 100 *μ*L/well were approximately 0.4 mM and 0.8 mM, respectively. Notably, the extent of cytotoxicity with both fresh and 4-h-decomposed SIN-1 was dependent on the volume of the medium per well; decreasing the volume in a well from 100 *μ*L to 50 *μ*L resulted in a proportional decrease in the cytotoxicity. Besides the LDH leakage assay, similar potent cytotoxic effect of 4-h-decomposed SIN-1 was also confirmed by MTT assay, an index of cellular metabolic activity [15] (Figure 3(b)). In addition, after 24 h the cytotoxicity of the decomposed SIN-1 in this solution was almost negligible (Figure 3(c)). To address whether the cytotoxicity of decomposed SIN-1 was specific for NaHCO_3_, SIN-1 was decomposed in 10 mM NaOH for 4 h, and cytotoxicity was assayed as described above (Figure 3(d)). HPLC analysis confirmed the complete conversion of SIN-1 to SIN-1C (data not shown). Surprisingly, essentially no cytotoxicity was observed for SIN-1 decomposed in NaOH, suggesting an important role of HCO_3_^−^ in SIN-1 cytotoxicity after decomposition. To further ascertain the requirement of HCO_3_^−^ in decomposed SIN-1 cytotoxicity, the concentration dependence of HCO_3_^−^ was measured. As shown in Figure 3(e), near-maximum cytotoxic potency was observed when SIN-1 (1 mM) was decomposed for 4 h in NaHCO_3_ in the concentration range from 2.5 to 40 mM. 

### 3.3. Decomposed SIN-1 in Bicarbonate Is Chemically Inert

**Figure 4:**
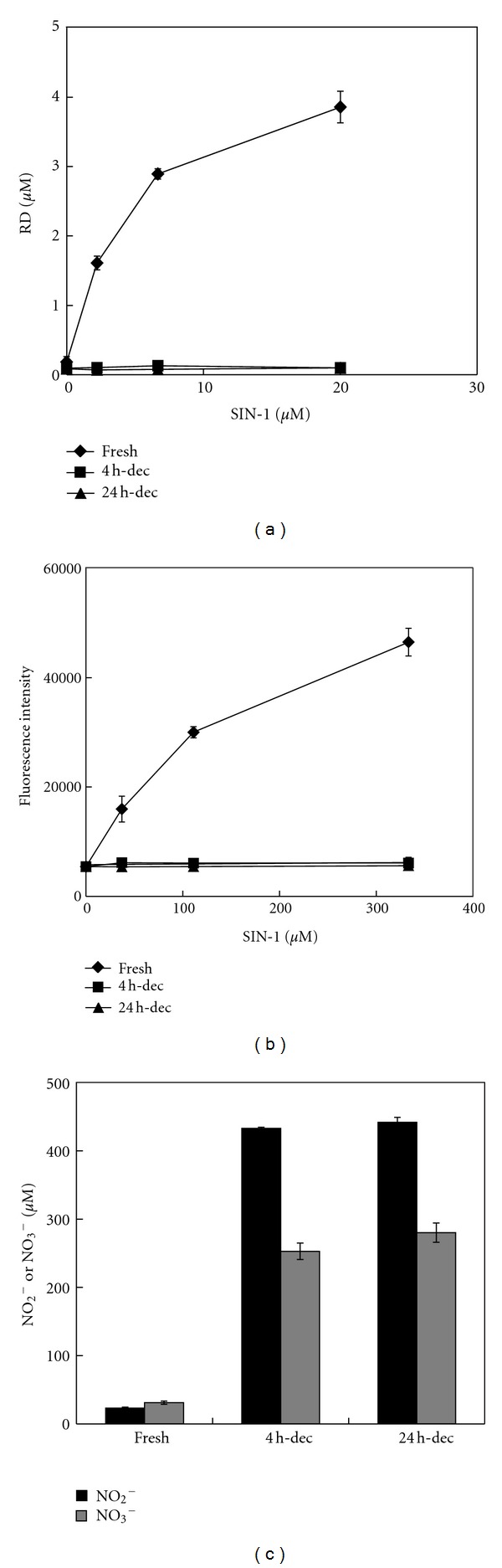
Chemical properties of decomposed SIN-1 in bicarbonate. (a) DHR-oxidizing activity. Serially diluted SIN-1 in 10 mM NaHCO_3_ was decomposed for 0, 4, and 24 h, neutralized with PBS, and incubated with DHR (final concentration 10 *μ*M) for 2 h. RD concentrations were calculated from a standard curve prepared from serially diluted RD. (b) N-nitrosating activity. Samples prepared as in (a) were mixed with DAN (final concentration 15 *μ*M) for 2 h, and the fluorescence intensity was measured. (c) Levels of NO_2_^−^ and NO_3_^−^ in each sample. The concentrations of SIN-1 in (a) and (b) represent the final concentrations in each assay. The concentrations of NO_2_^−^/NO_3_^−^ in (c) indicate their concentrations in the original SIN-1-decomposed NaHCO_3_ solutions. All the values shown are means ± intra-assay deviations, expressed as SD, from three wells in a representative experiment.

To gain insight into the cytotoxic mechanism, the oxidative and nitrosative activities of SIN-1 were measured. SIN-1 (1 mM) in 10 mM NaHCO_3_, either freshly prepared or decomposed for 4 h or 24 h in advance, was mixed with DHR, and RD formation was measured. As shown in Figure 4(a), fresh SIN-1 oxidized DHR in a concentration-dependent manner. However, after decomposition for more than 4 h, it did not oxidize DHR at all. The N-nitrosating activities of the same samples were evaluated with the DAN assay. Although fresh SIN-1 induced N-nitrosation of DAN in a concentration-dependent manner, neither 4-h- nor 24-h-decomposed SIN-1 caused DAN N-nitrosation (Figure 4(b)). Next, the NO_2_^−^ and NO_3_^−^ concentrations in these samples were compared (Figure 4(c)). Small amounts of both NO_2_^−^ and NO_3_^−^ were detected from freshly prepared SIN-1; however, these probably resulted from SIN-1 decomposition during the detection assay. In contrast, almost the same levels of NO_2_^−^ and NO_3_^−^ were detected in the cytotoxic 4 h SIN-1-decomposed NaHCO_3_ solution and in the innocuous 24 h SIN-1-decomposed NaHCO_3_ solution (Figure 4(c)). We also assessed ferrous cytochrome c oxidation and ferric cytochrome c reduction activities spectrophotometrically. Fresh SIN-1 showed potent oxidizing activity toward ferrous cytochrome c, but no reducing activity with ferric cytochrome c. Neither activity was detected with 4-h-decomposed SIN-1 (data not shown). Collectively, these data demonstrate that although significant cytotoxicity is retained, 4-h-decomposed SIN-1 in NaHCO_3_ is chemically inactive.

### 3.4. Cytotoxic Activity of SIN-1-Decomposed NaHCO_3_ is Thiol Sensitive

**Figure 5:**
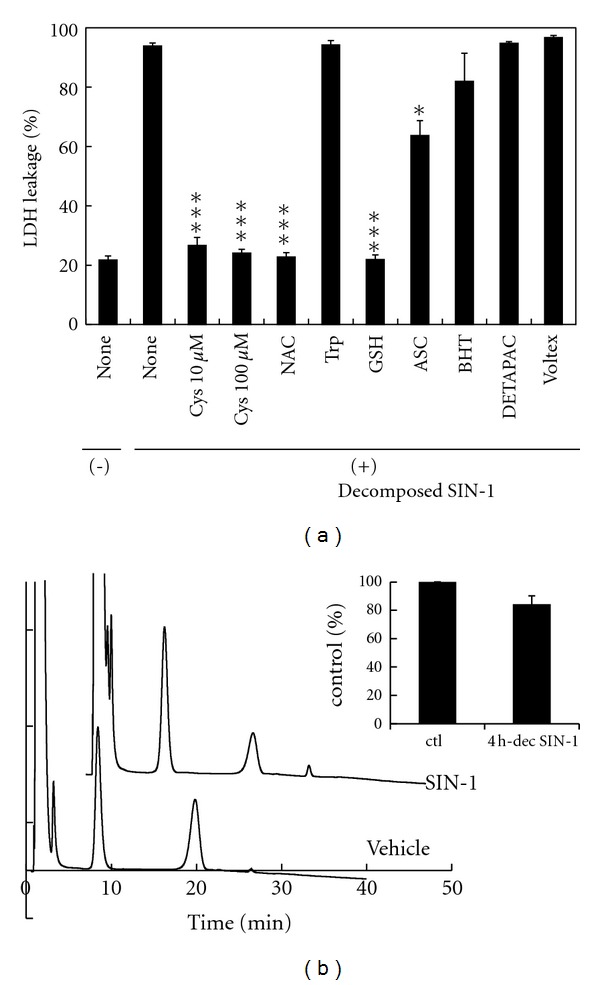
Effects of thiols on the cytotoxicity of 4-h-decomposed SIN-1 in bicarbonate. (a) Effects of amino acids, antioxidants, metal chelators, and air saturation on the cytotoxicity of decomposed SIN-1. Cells were treated with 4-h-decomposed SIN-1 (1 mM) prepared in NaHCO_3_ in conjunction with the indicated compound. The concentration for each compound was 100 *μ*M unless otherwise specified. The far-right column represents 4-h-decomposed SIN-1 (1 mM) saturated with oxygen by voltex mixing aerobically for 5 min. The values are means ± intra-assay deviations, expressed as SD, from four or five wells in a representative experiment. ***P < 0.001 versus decomposed SIN-1 alone. (b) HPLC profile of GSH after incubation with SIN-1-decomposed bicarbonate. GSH (50 *μ*M) was added to 4-h-decomposed SIN-1 (1 mM) in NaHCO_3_ or to the control NaHCO_3_ and incubated in a CO_2_ incubator for 2 h. Glu-Glu was added to the samples as an internal standard. The samples were derivatized and separated by HPLC on an NH_2_ column. GSH eluted at 22 min, GSSG at 27 min, and Glu-Glu at 12 min. The inset graph shows the GSH levels normalized to the area for Glu-Glu. The values are means ± inter-assay deviations, expressed as SD, from four independent assays. Note that the GSH level is consistently lower in the decomposed SIN-1 medium than in the control medium.

Our previous study demonstrated that the cytotoxicities of fresh SIN-1 and SIN-1 decomposed in FCS/RPMI were abolished by the addition of thiol compounds [8]. Therefore, the thiol sensitivity of the cytotoxic entity in 4 h SIN-1-decomposed NaHCO_3_ was examined. The addition of any thiol compound at 100 *μ*M, including L-Cys, NAC, and GSH, (Figure 5(a)) and D-Cys (data not shown), to 4-h-decomposed SIN-1 (1 mM) immediately prior to cell treatment almost completely abolished the cytotoxicity, whereas 100 *μ*M Trp had no effect (Figure 5(a)). Other than Cys, none of the amino acids tested, including Gly, Tyr, and Thr showed cytoprotection (data not shown).

To investigate whether the protective effect of thiols was dependent on their antioxidant activity and/or other properties, such as formation of coordination compounds due to Lewis base activity, the effects of ascorbate (ASC), the phenolic antioxidant butylated hydroxytoluene (BHT), and the metal chelator DETAPAC were measured (Figure 5(a)). Although ASC showed some protective effect, BHT and DETAPAC had no effect. We also assessed the effect of air resaturation prior to cell treatment, since oxygen depletion resulting from SIN-1 decomposition was a possibility. Vigorous aerobic voltex mixing of the SIN-1-decomposed NaHCO_3_ solution for 5 min before applying to cells had no effect on the cytotoxicity (Figure 5(a)). This result also suggested that the cytotoxic entity of 4-h-decomposed SIN-1 was stable to oxygen. The cytotoxic substance(s) was pH stable, since acidification of 4 h SIN-1-decomposed NaHCO_3_ with 20 mM HCl for 30 min followed by neutralization with 20 mM NaOH or the reverse (alkalization followed by neutralization) had no effect on its cytotoxicity (data not shown).

Because any thiol compound, including GSH, could reverse the cytotoxicity of the SIN-1-decomposed NaHCO_3_ solution (Figure 5(a)), there was a possibility of some modification of the thiol moiety. To address this possibility, changes in the GSH levels with and without incubation in 4 h SIN-1-decomposed NaHCO_3_ were measured by HPLC (Figure 5(b)). Incubation in SIN-1-decomposed NaHCO_3_ consistently decreased the GSH concentration by approximately 15% as compared with incubation in the control NaHCO_3_ solution (Figure 5(b), inset). However, although a very small increase in the GSSG peak was occasionally, but not consistently, detected, essentially no concomitant increase in the GSSG peak or emergence of novel peaks for GSH derivatives was observed under the HPLC conditions.

### 3.5. SIN-1 Cytotoxicity in Cell-Culture Medium Depends on the Total Amount of Initial SIN-1 Molecules per Cell, Irrespective of the Decomposition State

**Figure 6:**
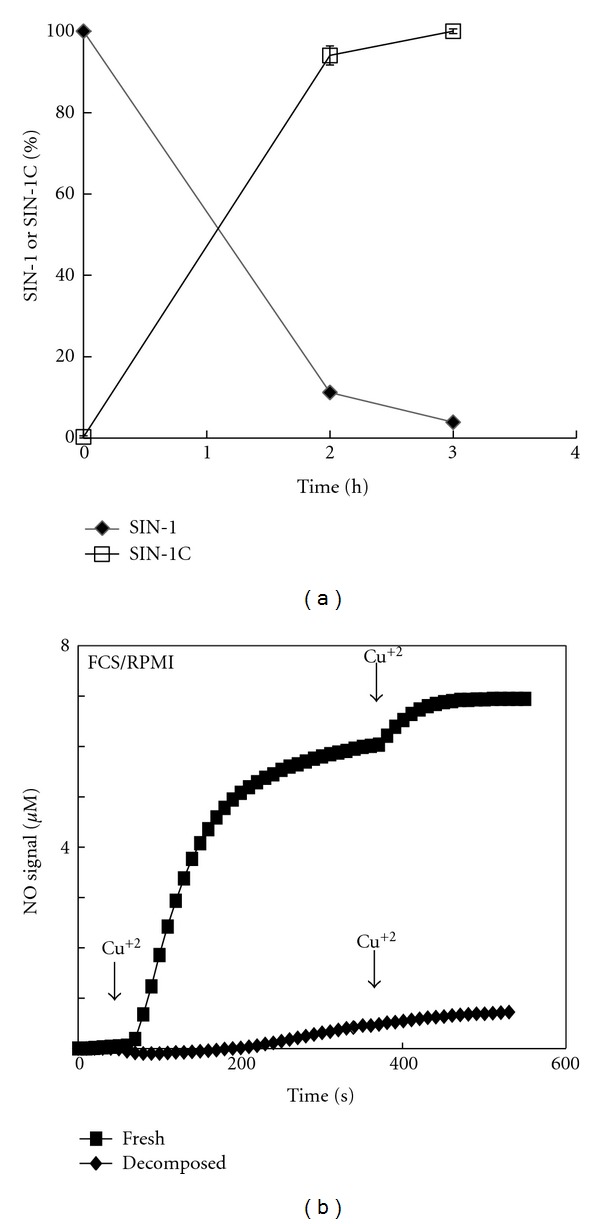
Decomposition of SIN-1 in FCS/RPMI. (a) Confirmation of SIN-1 decomposition. SIN-1 (1 mM) dissolved in FCS/RPMI was decomposed aerobically in a CO_2_ incubator for 2-3 h. At the indicated time points, serum proteins were precipitated with PCA, and samples were analyzed by HPLC, as described in Figure 2. The areas for SIN-1 at 0 h and SIN-1C at 3 h were taken as 100%. Values are means ± ranges of two independent experiments. (b) NO release from residual SIN-1 in the medium. The aliquot was diluted 10-fold with PBS, and NO levels were measured by an NO electrode. Where indicated by the arrows, CuSO_4_ was added to a final concentration of 1 mM to oxidize SIN-1.

To assess whether the cytotoxic entity of SIN-1-decomposed NaHCO_3_ was identical to that of SIN-1 decomposed in FCS/RPMI in the CO_2_ incubator [8], characteristics thus far observed for the cytotoxicity of SIN-1-decomposed NaHCO_3_ were assessed in the FCS/RPMI system. We first reassessed the extent of SIN-1 decomposition in FCS/RMPI by HPCL analysis. As shown in Figure 6(a), approximately 90% of the initial SIN-1 had been converted to SIN-1C after 2 h in a CO_2_ incubator. Next, we measured the level of NO released by the addition of Cu^2+^ to residual SIN-1 or S nitrosothiols that potentially had formed in serum proteins during SIN-1 decay in the medium [23, 24]. SIN-1 could be oxidized to its cation radical by oxidants other than O_2_ in the solution, thereby releasing only NO [11]. When freshly prepared SIN-1 (1 mM) in FCS/RPMI was diluted in PBS and oxidized with Cu^2+^ at a 10-fold molar excess, a significant level of NO liberation was observed (Figure 6(b)). Successive additions of the same concentration of Cu^2+^ resulted in slightly additive increases in the NO levels, suggesting that a significant portion of the original SIN-1 had been decomposed. In contrast, after a 2 h incubation in a CO_2_ incubator, the level of Cu^2+^-induced NO liberation from SIN-1 in FCS/RPMI was less than 10% of that from fresh SIN-1. Additional Cu^2+^ caused only a marginal increase. There results reconfirmed that SIN-1 decomposed significantly (>90%) within 2 h in FCS/RPMI in a CO_2_ incubator, consistent with our previous assessments by the Greiss and luminol chemiluminescence assays [8].

**Figure 7:**
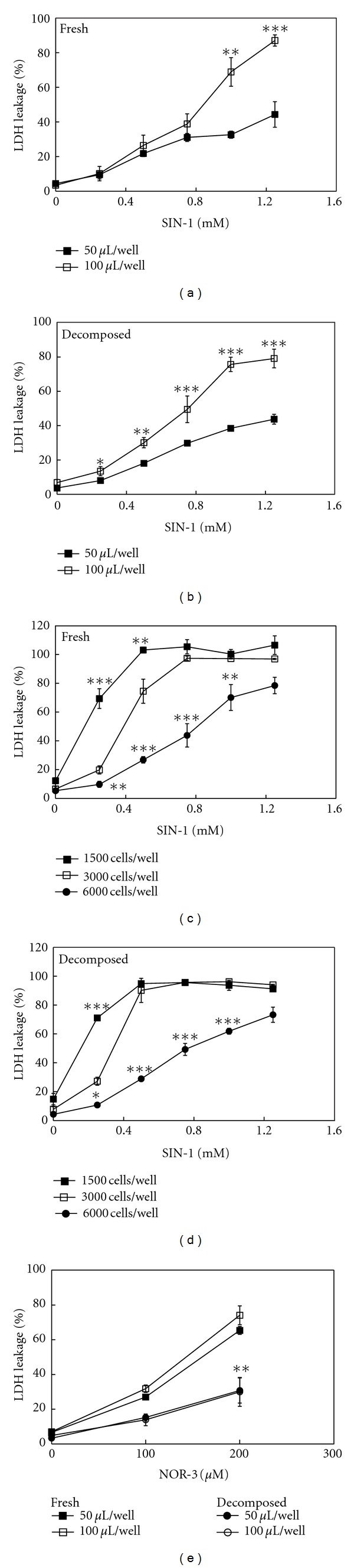
Dependence of SIN-1 cytotoxicity on the total number of initial SIN-1 molecules. (a and b) Treatment volume of medium dependence of SIN-1 cytotoxicity on PC12 cells. Cells in 96-well plates (3000 cells/well) were treated with the indicated concentrations of either freshly prepared SIN-1 in FCS/RPMI (a) or 2 h SIN-1-decomposed FCS/RPMI (b) at either 50 *μ*L/well or 100 *μ*L/well for 24 h. The cytotoxicity was evaluated by LDH leakage (%). *P < 0.05, **P < 0.01, and ***P < 0.001 versus respective control. (c and d) Inverse correlation between SIN-1 cytotoxicity and cell density. Cells cultured at different initial densities (1500, 3000, or 6000 cells/well) were treated with the indicated concentrations of freshly prepared SIN-1 in FCS/RPMI (c) or 2 h SIN-1-decomposed FCS/RPMI (d) at 100 *μ*L/well for 24 h. *P < 0.05, **P < 0.01 and ***P < 0.001 versus respective 3000 cells/well. (e) Characteristics of NOR-3 cytotoxicity. Cells in 96-well plates (3000 cells/well) were treated for 24 h with the indicated concentrations of freshly prepared NOR-3 in FCS/RPMI or 2 h NOR-3-decomposed FCS/RPMI at either 50 *μ*L/well or 100 *μ*L/well. Cytotoxicity was measured after 24 h. The values are means ± intra-assay deviations, expressed as SD, from four or five wells in a representative experiment. **P < 0.01 versus, fresh NOR-3.

Using fresh SIN-1 and 2 h decomposed SIN-1 in FCS/RPMI, the volume dependency of SIN-1 cytotoxicity was assessed. Cells were treated with increasing concentrations of freshly prepared SIN-1 in FCS/RPMI with different volumes per well. Treatment at 100 *μ*L per well resulted in cells dying with an LD_50_ of about 0.5 mM (Figure 7(a)), consistent with our previous results [8]. Surprisingly, however, when cells were treated with the same concentrations of SIN-1 but with half the volume of medium (50 *μ*L per well), cytotoxicity was proportionately decreased. An almost identical result was obtained with 2 h decomposed SIN-1 in FCS/RPMI (Figure 7(b)). To clarify whether the medium-volume dependence of SIN-1 cytotoxicity could have resulted from the difference in the height of the medium over the cells or from the absolute number of SIN-1 molecules per cell, different cell densities (1500, 3000, or 6000 cells/well) were treated with fresh or decomposed SIN-1 in 100 *μ*L of medium. As shown in Figures 7(c) and 7(d), the extent of the cytotoxicity of fresh and decomposed SIN-1 decreased inversely with increasing cell density; the LD_50_s in both fresh and decomposed SIN-1 at 1500, 3000, and 6000 cells/well were approximately 0.2, 0.4, and 0.8 mM, respectively. These results suggested that both fresh and decomposed SIN-1 cytotoxicity was dependent on the total number of initial SIN-1 molecules per cell, rather than on the concentration.

To examine whether these characteristics were peculiar to SIN-1 cytotoxicity, similar experiments were performed on NO-induced cytotoxicity. NOR-3 was employed as an NO donor because its half-life is approximately 30 min at 37°C [25], which is close to that of SIN-1. NOR-3 killed cells in a concentration-dependent manner up to 200 *μ*M (Figure 7(e)). In contrast to the results with SIN-1, the cytotoxicity curves for NOR-3 at 50 *μ*L/well and 100 *μ*L/well were nearly superimposable. Moreover, after decomposition for 2 h in FCS/RPMI, the extent of cytotoxicity decreased to less than 40% of the freshly prepared counterpart. These results suggested that NO cytotoxicity from NOR-3 was reasonably dependent on the concentration of the remaining NO donor molecules at a particular time point, in stark contrast to the unusual toxicological characteristics of SIN-1.

### 3.6. FCS Is Not Essential for the Cytotoxicity of Decomposed SIN-1 in FCS/RPMI

SIN-1 retained its cytotoxicity after decomposition in a simple solution of NaHCO_3_ (Figure 3).

**Figure 8:**
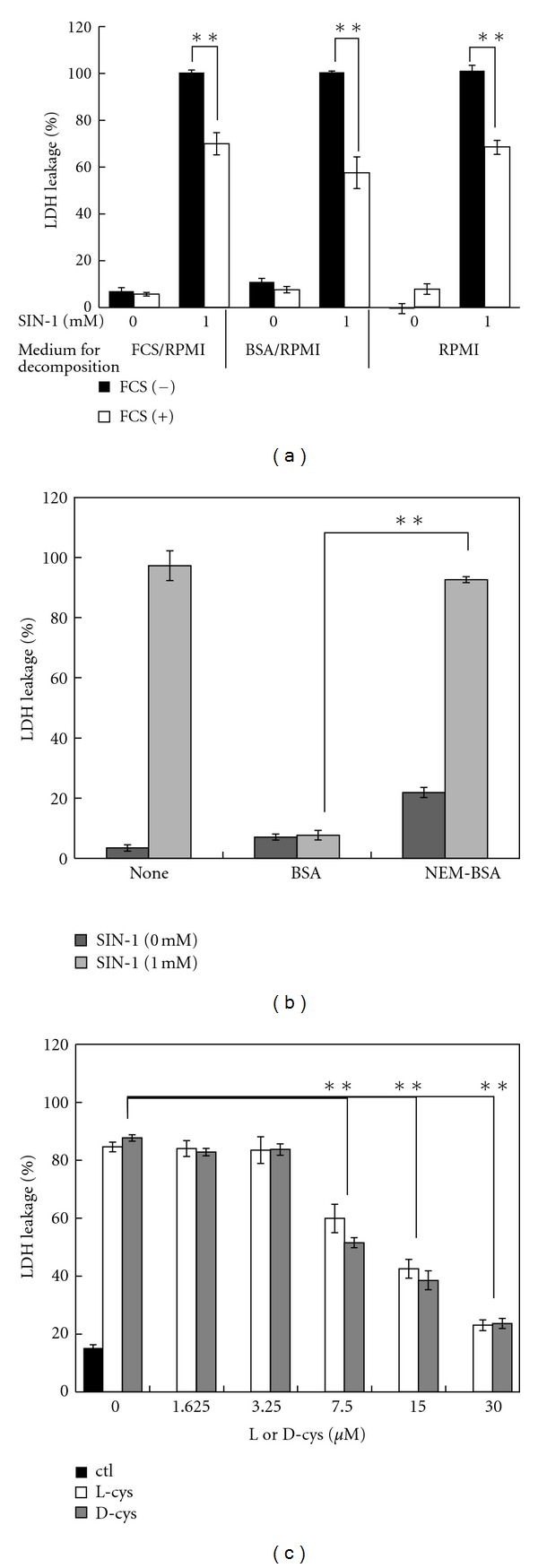
Effects of FCS, BSA, and Cys on the cytotoxicity of decomposed SIN-1 in cell culture media. (a) Effect of FCS on the cytotoxicity of decomposed SIN-1. SIN-1 (1 mM) was decomposed in either 10% FCS/RPMI, BSA (3 mg/mL)/RPMI, or RPMI for 2 h as in shown in Figure 7, followed by the addition of fresh FCS at a final concentration of 10% and exposure to cells at 100 *μ*L/well. Viability was measured 24 h after exposure. (b) Comparison of the protective effects of BSA and NEM-BSA against the cytotoxicity of 2 h SIN-1-decomposed FCS/RPMI. Cells were treated with 2 h SIN-1-decomposed FCS/RPMI (1 mM), together with BSA or NEM-BSA (3 mg/mL). (c) Effect of Cys on the cytotoxicity of decomposed SIN-1. As in (b), cells were treated with 2 h SIN-1-decomposed FCS/RPMI (1 mM) together with the indicated concentrations of D- or L-cysteine. All values are means ± intra-assay deviations, expressed as SD, from four or five wells in a representative experiment. **P < 0.001.

Contrary to these results, we demonstrated previously that the presence of FCS during SIN-1 decomposition was indispensable for the manifestation of cytotoxicity by 2 h-decomposed SIN-1 in RPMI [8]. In that experiment, however, cells were exposed to a combination of SIN-1-decomposed RPMI and fresh FCS (final concentration 10%) and the cytotoxic effect were compared with that of SIN-1-decomposed FCS/RPMI. Therefore, we reexamined the requirement of FCS in the cytotoxicity of 2 h SIN-1-decomposed medium. SIN-1 (1 mM) was decomposed in either FCS/RPMI, RPMI containing 3 mg/mL BSA (BSA/RPMI; equivalent in protein concentration to 10% FCS), or RPMI, and exposed to cells with or without supplementation of fresh FCS (final concentration 10%) immediately before exposure. Without the addition of fresh FCS, significant cytotoxicity was observed with all SIN-1-decomposed media, including RPMI (Figure 8(a)). However, the addition of fresh FCS resulted in partial (approximately 30%) attenuation of the cytotoxicity in all SIN-1-decomposed media. These results demonstrate that SIN-1-decomposed medium retains cytotoxicity regardless of the presence of FCS or BSA during the decomposition period, and that fresh FCS contains substances that can antagonize the cytotoxicity of decomposed SIN-1 when added after SIN-1 decomposition.

Because the principle proteinous constituent of FCS is BSA, we measured the effect of BSA supplementation on the cytotoxicity of 2 h SIN-1-decomposed FCS/RPMI. Addition of BSA at 3 mg/mL before exposure of cells almost completely suppressed the cytotoxicity of SIN-1-decomposed FCS/RPMI (Figure 8(b)). BSA has 35 cysteine residues, of which 34 are involved in intra-molecular disulfide bridges, and half of the one remaining is blocked by a free cysteine via a disulfide bond [26]. To gain insight into the cytoprotective role of BSA against SIN-1-decomposed media, we blocked the free Cys in BSA with N-ethylmaleimide (NEM). Interestingly, NEM-treated BSA completely lost its cytoprotective activity, demonstrating that free-sulfhydryl groups in BSA play a critical role in cytoprotection against decomposed SIN-1. Thus, these results clarified that the presence of FCS during SIN-1 decomposition is not essential for the cytotoxicity of decomposed SIN-1 in cell-culture medium.

Previously, we showed that L cysteine and GSH could attenuate the cytotoxicity of fresh SIN-1 and SIN-1-decomposed FCS/RPMI [8]. To gain an insight into the protective mechanism of thiols, the effects of L-Cys and D-Cys, and the biologically active and inactive forms, respectively, were compared. Both cysteine isomers protected cells from 2 h SIN-1-decomposed FCS/RPMI with the same concentration dependence and an LD_50_ of approximately 20 *μ*M (Figure 8(c)). Overall these results clearly demonstrate that the cytotoxicity of decomposed SIN-1 in FCS/RPMI is sensitive to thiols.

### 3.7. SIN-1 Cytotoxicity Involves Mitochondrial Damage and GSH Depletion, Irrespective of the Decomposition State

**Figure 9:**
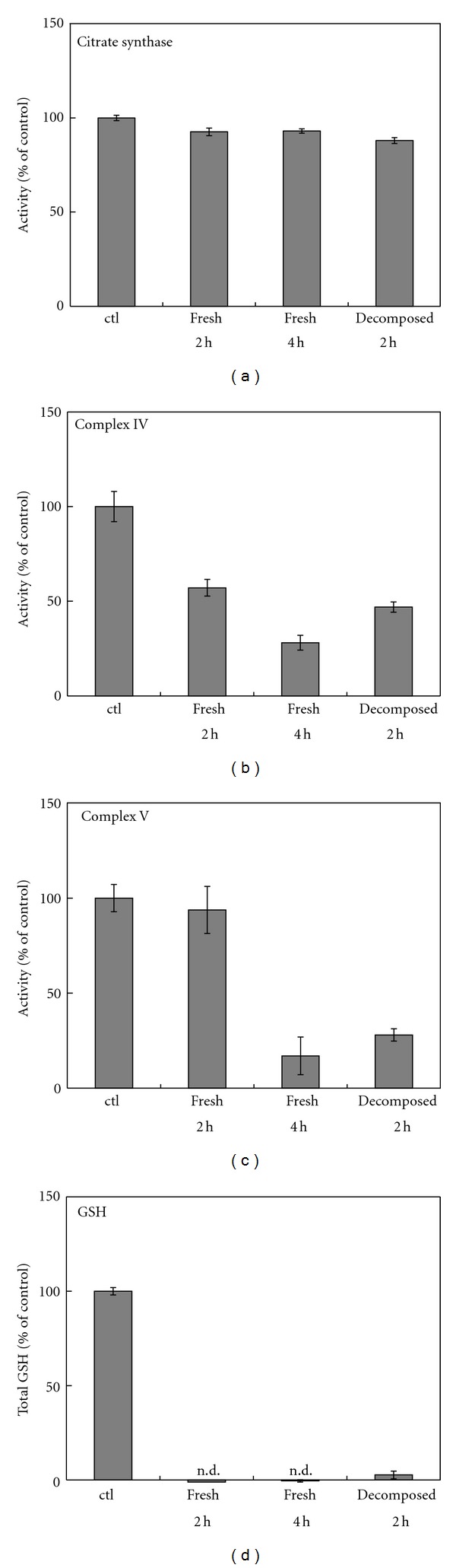
Effect of SIN-1 treatment on GSH content and mitochondrial respiratory enzyme complex activities. Cells in 6-mm dishes were treated with fresh SIN-1 in FCS/RPMI or 2 h SIN-1-decomposed FCS/RPMI (1 mM) for 2 h or 4 h, and the activities of citrate synthase (a; control activity = 114 mU/mg), complex IV (b; control activity = 7.9 k/mg), and complex V (c; control activity = 11.6 mU/mg), and the GSH content (d; control cells, 11.9 nmol/mg) were measured. Values are means ± ranges from two wells in a representative experiment and are expressed as a percentage of the control. n.d.: not detectable.

It is well documented that NO treatment, specifically at high concentrations or with persistent treatment, leads to inactivation of the enzyme complexes of the electron transport chain in mitochondria [20, 27]. To examine whether the cytotoxicities of fresh and decomposed SIN-1 were also associated with mitochondrial damage preceding the onset of cell death, mitochondrial enzyme activities were measured. Our previous study had revealed that with fresh SIN-1, LDH leakage occurred approximately 5 h after exposure, whereas, with decomposed SIN-1, it was evident as early as 3 h after exposure [8]. Therefore, mitochondrial enzyme activities were measured at 2 h and 4 h after exposure to freshly prepared SIN-1 in FCS/RPMI and at 2 h after exposure to 2 h SIN-1-decomposed FCS/RPMI. Neither the fresh nor decomposed SIN-1 affected complex I activity (data not shown) or citrate synthase activity (Figure 9(a)). In contrast, both the fresh and decomposed SIN-1 reduced complex IV (cytochrome c oxidase) activity to approximately 50% of the control after a 2 h exposure (Figure 9(b)). Although no decrease in complex V (ATP synthase) activity was observed with fresh SIN-1 treatment for 2 h, more than 70% of the activity was lost by treatment with decomposed SIN-1 for 2 h (Figure 9(c)). However, by 4 h, fresh SIN-1 had also reduced complex V activity by more than 70%. Overall, these results suggest that SIN-1 imposes mitochondrial damage regardless of its decomposition state and that decomposed SIN-1 has the potential to induce damage faster than fresh SIN-1. 

The effects of fresh and decomposed SIN-1 treatments on cellular antioxidants and antioxidative enzymes were measured. Exposure to fresh SIN-1 almost completely depleted cellular GSH as early as 2 h after exposure (Figure 9(d)). Similarly, exposure to decomposed SIN-1 resulted in the complete depletion of GSH within 2 h. In contrast, none of the antioxidant enzymes examined, including NAD(P)H quinone oxidoreductase (NQO-1), GSH reductase (GR), or superoxide dismutase (SOD, total), was affected by either fresh or decomposed SIN-1. These results suggest that SIN-1, irrespective of its fresh or decomposed state, selectively depletes the GSH content of the cells.

## 4. Discussion

The present study demonstrated that although SIN-1 completely decomposed within 2 h in NaHCO_3_ at 37°C (Figure 2), significant cytotoxicity was stably present in the SIN-1-decomposed NaHCO_3_ after an additional 2 h and that the extent of cytotoxicity was dependent on the volume of the treatment medium (Figure 3(a)). In contrast, when SIN-1 was decomposed for the same period in NaOH, essentially no cytotoxicity was observed (Figure 3(c)), indicating a critical role for HCO_3_^−^ in the formation of cytotoxic substance(s). Although the cytotoxicity of SIN-1-decomposed NaHCO_3_ declined significantly by 24 h (Figure 3(b)), no substantial difference was observed between 4 h and 24 h decomposed SIN-1 in the HPLC profiles of SIN-1 breakdown products monitored at 290 nm (Figure 2) or 210 nm (data not shown). Analysis of the chemical properties of the 4 h SIN-1-decomposed NaHCO_3_ demonstrated a complete lack of either oxidizing or nitrosating potential, similar to its innocuous 24 h decomposed counterpart (Figures 4(a) and 4(b)). One notable feature of the cytotoxic entity of 4 h SIN-1-decomposed NaHCO_3_ was its thiol sensitivity (Figure 5). Nevertheless, modifications of thiol after incubation with 4 h SIN-1-decomposed NaHCO_3_ were marginal (Figure 6). 

### 4.1. Involvement of ONOOCO^−^ in SIN-1 Cytotoxicity

**Figure 10:**
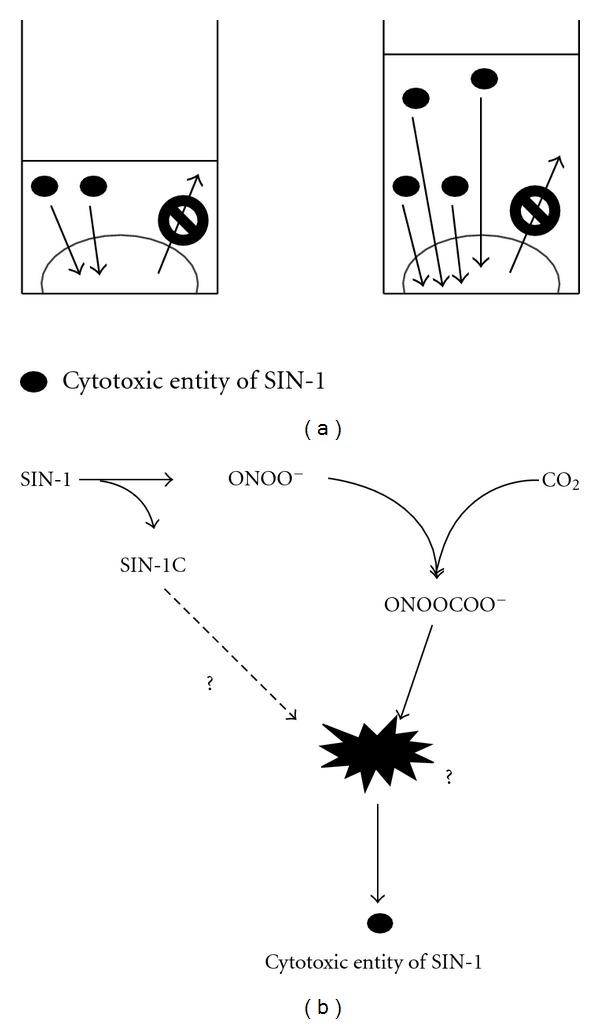
Proposed mechanism of SIN-1 cytotoxicity. (a) Mechanism of the dependence of SIN-1 cytotoxicity on the total number of initial SIN-1 molecules per cell. SIN-1 decomposition yields a stable cytotoxic entity, which enters the cells and is promptly metabolized, resulting in cytotoxicity. Because of the rapid metabolism, cellular incorporation of the cytotoxic entity of SIN-1 is one directional, and therefore the cytotoxic effect is augmented when the volume of SIN-1-containing medium is increased with respect to the number of cells. (b) Potential cytotoxic entity of SIN-1. SIN-1-derived ONOO^−^ reacts with CO_2_ in the culture medium, resulting in the formation of ONOOCO_2_^−^. A small portion of the ONOOCO_2_^−^ thus generated, or its derivatives, may be stabilized by forming a complex with SIN-1C or other SIN-1-derived breakdown products, which renders the complex chemically inert. However, interaction with cells converts it to cytotoxic substances.

The dependence of SIN-1 cytotoxicity on the volume of the treatment medium at a particular concentration, regardless of its fresh or 4-h-decomposed state (Figure 3(a)), suggests that the toxicant concentration in the medium continues decreasing and never reaches equilibrium with its cellular targets (Figure 10(a)). In other words, the toxicant concentration in the medium is effectively low enough to decline promptly in a manner dependent on the presence of cells; that is, cellular incorporation of the toxicant is a rapid and irreversible process, such as one involving covalent binding or rapid metabolism although the toxicant itself is relatively stable (Figure 3(c)).

The requirement for NaHCO_3_ in the cytotoxicity of decomposed SIN-1 (Figure 3) undoubtedly indicates the incorporation of HCO_3_^−^ into the toxicant. In aqueous solution, HCO_3_^−^ releases CO_2_ as follows:

(1) 2HCO3−  ⟷  CO32−  +  H2O  +  CO2

It is well documented that ONOO^−^ can react with CO_2_ to form nitrosoperoxocarboxylate (ONOOCO_2_^−^) [28, 29] as follows:

(2) ONOO−  +  CO2  →  ONOOCO2−

Therefore, the difference between NaHCO_3_ and NaOH with regard to cytotoxicity is possibly the formation of ONOOCO_2_^−^, at least, at an initial stage (Figure 10(b)). ONOOCO_2_^−^ is suggested to readily undergo hemolytic fission, yielding the potent oxidant carbonate radical (CO_3_^.−^) and NO_2_ radical (NO_2_^.^) or to rearrange and decompose into NO_3_^−^ and CO_2_ [28, 29]. Because decomposed authentic ONOO^−^ is not cytotoxic [8], breakdown product(s) of SIN-1 could also be involved in the toxicant and, unless cells are present, render the complex stable and chemically inactive (Figures 4(a) and 4(b)). Taken together, one potential candidate for the toxicant in decomposed SIN-1 may be a small amount of ONOOCO_2_^−^ or its derivatives that are concurrently stabilized by interaction with other molecules; that is, the cationic nature of protonated SIN-1C. Because ONOOCO_2_^−^ can take either inert, *cis*, or reactive *trans* rotameric states [30, 31], it may also be possible that either rotamer is selectively incorporated into the toxicant. The antagonizing effect of thiols without significant thiol modification (Figure 5) suggests that thiols may disrupt the interaction of ONOOCO_2_^−^ or its derivatives with a putative stabilizer.

### 4.2. Bicarbonate-Dependent Toxicant Formation May Be a General Mechanism for SIN-1 Cytotoxicity

In FCS/RPMI, approximately 90% of SIN-1 was converted to SIN-1C in 2 h (Figure 6). Nevertheless, similar to the case with NaHCO_3_ (Figure 3), the 2 h SIN-1-decomposed medium was as cytotoxic as its freshly prepared counterpart (Figure 7) [8]. Given that HCO_3_^−^ is a prime buffering component of FCS/RPMI maintained in a CO_2_ incubator and that the cytotoxic characteristics of SIN-1-decomposed FCS/RPMI are shared with those of SIN-1-decomposed NaHCO_3_, including the reciprocal relationship between cell density and the severity of toxicity (Figure 7) and thiol sensitivity (Figure 8), the same toxicant from SIN-1-decomposed NaHCO_3_ could be responsible for the cytotoxicity of 2 h SIN-1-decomposed FCS/RPMI.

We further propose that the HCO_3_^−^-dependent cytotoxic substance may be responsible for SIN-1 cytotoxicity in general; that is, treatment with freshly prepared SIN-1. During cell treatment with SIN-1 in culture medium in a CO_2_ incubator, the cytotoxic substance could accumulate in the medium, which in turn exerted cytotoxicity. Fresh SIN-1 induced an approximately 2 h delay in LDH leakage [8] and a 2 h delay in mitochondrial damage, particularly to complex V (Figure 9(c)), than decomposed SIN-1, thus supporting our assumption. The association of severe GSH depletion with treatment by either fresh or decomposed SIN-1 (Figure 9(d)) and the sensitization to fresh and decomposed SIN-1 cytotoxicity by prior GSH depletion with buthionine sulfoximine (BSO) treatment [8] suggest that oxidative and/or nitrosative stress is involved during the SIN-1-induced cell-death process. Because depletion of GSH [32], mitochondrial damage [20, 27], and the sensitizing effect of BSO [33] are commonly observed in NO-induced cytotoxicity, the HCO_3_^−^-dependent toxicant of SIN-1 may liberate NO or its derivatives on contact with cells. 

Previously, Li et al. [34] reported that SIN-1 cytotoxicity to lymphoblastoid cells treated in Hank's balanced salts supplemented with bicarbonate showed an inverse correlation between cell density and the severity of the cytotoxicity, as we demonstrated using PC12 cells, suggesting that the above cytotoxic scheme may be applicable to other cell types. Thus, identifying the cytotoxic entity of SIN-1, as well as its in-depth cytotoxic mechanism, is imperative for research using SIN-1. Further research should be directed at conclusively identifying this elusive entity.

## 5. Conclusions

The present study demonstrates that SIN-1 cytotoxicity is maintained even after its complete decomposition in medium containing bicarbonate. ONOOCO_2_^−^ is a potential key intermediate for the formation of the toxicant. The toxicant induces cytotoxicity that is dependent on the absolute number of initial SIN-1 molecules per cell. Although the toxicant is chemically inert, it inflicts damage to cells in a manner similar to NO-related stress. Thus, careful interpretation is necessary for data obtained with SIN-1.
